# Surgery

**Published:** 1876-12

**Authors:** 


### III. Surgery.

1. *Traumatic Tetanus cured by Nerve-stretching.* Vogt. (*Centralbl. f. Chir.*, No. 40, 1876.)

Since the publication of Prof. Nussbaum's famous case of neuralgia cured by stretching the cervical plexus, the operation of nerve-stretching has been repeatedly performed by Nussbaum, Billroth, Callender, Vogt, and others. The operation was applied for the cure of neuralgia, clonic spasms and various other nervous affections. It always had the desired effect, and proved to be free from danger. These good results surely warranted the experiment of the operation in the following case: On August 23, a stone mason, aged sixty-three years, had his right hand wounded by a heavy stone. The large flap wound of the palm of the hand kindly healed under antiseptic dressings, while the smaller wound of the back of the hand very slowly healed by granulations. On September 7, the attending physician found the first evidence of trismus, and two days later tetanus supervened. On September 16, Prof. Vogt found the patient suffering from a continuous lock-jaw, and he was frequently seized with violent attacks of opisthotonos and clonic spasms, while the lower extremities were excessively rigid. Neither the recent thick cicatrix in the palm of the right hand, nor the surroundings of the well granulating wound at the back of the hand, showed any tenderness on pressure. No painful spots existed in the course of the nerves of the forearm or arm; but the region of the brachial plexus was so sensitive that the slightest touch produced convulsions. The patient, in spite of all treatment, had been daily growing worse; therefore the professor at once resorted to the traction upon the brachial plexus. First, however, the palmar cicatrix was cut into and freed from the aponeurosis and the tendons; a foreign body was not found. On the right side of the neck, then, a longitudinal incision was made, parallel to the anterior border of the trapezius muscle, and in the triangular space between the trapezius, omohyoid and scalenus muscles, the brachial plexus was exposed. The sheath of the nerves being severed, a blunt hook was passed under the nerves to lift them out of their surroundings. The flexed forefinger could there be substituted for the hook, and make energetic tractions on the central as well as the peripheric portions of the nerves. The sheath of the nerves, looking exceedingly hyperæmic, was split up as far as the intervertebral foramina. A drainage tube was put in, and the wound was dressed antiseptically. No sooner had the patient recovered from the narcosis than he could open his mouth sufficiently to put out his tongue and to take some fluid nourishment. The tetanic rigidity and spasms of the muscles had entirely disappeared and did not return, a few slight momentary twitchings in the neck and back excepted, which occurred on the patient's first sitting up in a chair. The patient felt exceedingly weak, and slept very much during the day, while at night morphia had to be administered for restlessness. No other medicine was used. And now, ten days after the operation, the wounds are nearly healed, and the patient can, *ad libitum*, stand and sit and walk; nor does he experience any disturbance of sensibility or motility in his right arm.

2. *Resection of the Superior Maxilla with Preservation of the Infra-orbital Nerve.* LETIEVANT. (*Archives Générales de Méd.*, Oct., 1876.)

The author recalls the idea expressed by Longet and other physiologists, that the sensitive filaments of nerves exercise a nutritive influence upon muscular fiber. The results of the operation of ablation of the superior maxilla, with preservation and destruction of the infra-orbital nerve, are compared:

1. Destruction of nerve. The results were simple, with the exception of a slight attack of intercurrent erysipelas. The patient left the hospital affected with slight œdema of the cheek, and preserving cutaneous sensibility. Eight months afterward the mobility of the upper lip was slight, and the patient could not puff both cheeks without the escape of air. Electrization of muscles through the skin did not secure contraction; and when the needles were implanted in the muscles there was no greater effect.

2. Preservation of nerve. This is readily accomplished, and does not add much to the time of duration of the operation, if the entire nerve is laid bare and elevated; the suborbital tissue is left attached to the deep portion of the zygoma. Thirteen months after the operation all the facial muscles contracted; sensibility, as determined by the æsthesiometer, was absolutely alike on both sides; pressure only did not produce the same sensation as before, in consequence of destruction of the anterior dental filaments. The restoration of the face was remarkable, thanks to the osseous tripod left by the surgeon; cicatrization had not deformed the visage; and, more, an inodular mass filled the hiatus left by the maxillary line.

3. *Treatment of Tracheal and Laryngeal Stenosis by Dilatation.* THAOU. (*Le Progrès Méd.*, No. 38.)

Syphilis, small-pox and typhoid fever, from their tracheal and laryngeal localizations, are the most frequent causes of contractions of the ærial conduit, and in these stenosis is most surely caused by submucous lesions. Perichondritis is almost invariably observed, and in its train occur necrosis of bone and anchylosis of the small articulations. It develops an exudation which leads to prominence of the mucous membrane, ulceration or fibrous transformation, compressed and therefore inactive muscles, which undergo fatty degeneration. These processes are hardly evident to the laryngoscope. Sometimes the epiglottis is destroyed and falls upon the glottis, where, becoming adherent, it remains fixed in a vicious position.

It must be elevated with a curved sound in order to uncover the completely obstructed glottis. It is surprising that respiration should be possible through such a tortuous and occluded canal. Sometimes the tube is closed by a true diaphragm, more or less excentrically perforated, and composed of a whitish cicatricial membrane.

The symptoms are referable to a distant date, when there was hoarseness, dyspnœa, and a sense of suffocation. Then tracheotomy does not cure; it saves life, but leaves behind the stricture which pre-existed, and a true fistule of a dangerous character. Existence at such a price is dear.

Before the tracheal tube can be removed, the natural passage-way of the air must be restored. Liston (1827), Czermak (1851), Güérin, Richet, Busch, Brake, Gurlt, Bruns, Dolbeau, Gerhardt, Treudelenburg, operated by instruments passed upward through the artificial opening.

The direct method (by the mouth) has been tried by only Roux, Desprès, Thiersch and Navratil — always without success.

After two months' trial with the method of Schrötter, the author is disposed to recommend it highly. The former operates by the superior or natural passage, but the method of operating and the form of his instruments vary according to whether there (*a*) is or (*b*) is not a surgical opening in the neck.

(*a*) In this case the tracheal fistula is utilized only for the purpose of fixing in the larynx a dilating prism, introduced through the mouth. The prisms are zinc, triangular in shape, with rounded borders, and resemble in form the glottis. Inferiorly they present a small narrowed neck, surmounted by an equally small rounded head. The prism descends into the air passage and becomes engaged in the opened tracheal cannula, toward its dome. A pair of slender forceps, capable of continuous pressure, is introduced through the neck, seizes the prism at its constricted part, and holds it in place. At its upper extremity the prism is provided with a stout thread. At the time of introduction this thread is made to pass through a long curved tube, when it is firmly fastened by a knot to the handle by which the tube is carried. Thus constituted, the apparatus represents an uninterrupted instrument, composed of a curved branch (the tube) and a terminal enlargement (the prism). By the aid of the laryngeal mirror, it is engaged in the larynx and fixed below by the tracheal forceps. The knot is then unfastened; the tube, which is no longer retained by the prism, is withdrawn; and the thread, which remains attached to the prism, is brought out of the mouth and attached to the ear. The patient or physician has but to draw upon the thread and open the forceps in order to extract the prism from the larynx.

Some education of the parts is requisite before proceeding to the dilatation, which is accomplished by the successive introduction of a series of prisms, numbered from one to twenty-four—the smallest being eight millimeters in one measurement and six in the other, the largest being twenty by sixteen. All are four centimeters long. The duration of the dilatation is from a few minutes at the outset to the entire night, the patient being finally able to sleep with the instrument *in situ*, because accustomed to its presence. The results are satisfactory to a surprising degree. Soon the knife or galvano-cauter may be used to divide a bridle, or various caustic applications may be made to localized swollen tissue. Sometimes the voice returns, or the patient can breathe and talk with the cannula closed. Insuccess is due generally to obstructions lower down. Of course more recent cases afford the best results.

(*b*) In non-tracheotomized subjects the sounds are adapted to the different conditions, and the patients learn to use them themselves. The base of the tongue is depressed with the finger, and the instrument made to penetrate the larynx without hesitation. Schrötter employs also rubber sounds, somewhat longer than those described above, with a triangular laryngeal extremity and rounded borders. Lateral openings are made for the exit of mucous secretions. There is also an oval shield of hard rubber, so arranged that the expectorated secretions are directed to the floor, and not in the face of the operator. Often a previous incision of the obturator membrane by the knife or cauter is requisite before the introduction of the sound.

4. *Diseases of the Nose treated by Medicated Bougies.* CATTI. (*Memorabilien*, 1876, No. 9.)

At a meeting of the Vienna Medical Society, Catti showed some gelatine bougies charged with astringent medicines—as sulphate of copper, zinc, carbolic acid, tincture of rhatanny. They are from eight to eleven centimeters long, and from four to six centimeters thick. By a slow rotatory motion they are introduced into the nasal cavity, and by giving them a horizontal or slanting or perpendicular direction they can be inserted into the lower or middle nasal passage. The bougie inserted, the nostril is plucked with charpie or cotton, and in a few hours it will be entirely melted. By a regular continued use of such bougies Catti obtained very good results in the treatment of chronic catarrh of the nose and naso-pharyngeal space and of the ozæna.

